# American Medical Association

**Published:** 1875-03

**Authors:** 


*American Medical Association.*

The Twenty-sixth Annual Session will be held in the city of Louisville, Ky., on Tuesday, May 4th, 1875, at 11 A. M.

"The Delegates shall receive their appointment from permanently organized State Medical Societies, and such County and District Societies as are recognized by representation in their respective State Societies, and from the Medical Department of the Army and Navy of the United States."

"Each State, County, and District Medical Society entitled to representation shall have the privilege of sending to the Association one delegate for every ten of its regular resident members, and one for every additional fraction of more than half that number: *Provided*, however, that the number of delegates for any particular State, Territory, County, City or Town shall not exceed the ratio of one in ten of the resident physicians who may have signed the Code of Ethics of the Association."

"The Chairmen of the several sections shall prepare and read in the general sessions of the Association, papers on the advances and discoveries of the past year in the branches of science included in their respective sections. * * *."—*By-Laws, Art. II, Sec.* 4.

"Papers appropriate to the several sections, in order to secure consideration and action, must be sent to the Secretary of the appropriate section at least one month before the meeting which is to act upon them. It shall be the duty of the Secretary to whom such papers are sent, to examine them with care, and, with the advice of the Chairman of his section, to determine the time and order of their presentation, and give due notice of the same. * * *"—*By-Laws, Art. II., Sec.* 5.

By Dr. Adams Jewett, Ohio:

"The Permanent Members shall consist of those who have served in the capacity of delegates, and of such other members as shall have received the appointment by unanimous vote, and of all others who, being members in good standing of any State or local Medical Society entitled to representation in this body, shall, after being vouched for by at least three members, be elected to membership by a vote of two-thirds of the delegates in attendance, and shall continue such so long as they remain in good standing in the body of which they were members when elected to membership in this Association, and comply with the requirements of its By-Laws."

We regret that, for the want of space, we cannot give the entire programme. We feel warranted in saying, however, from our knowledge of the high character and scientific attainments of the gentlemen composing the various sections and committees, that the approaching session will be, perhaps, the most profitable and interesting that has ever been held since its organization. We trust it will be largely attended by the great and good men of the profession.

